# Bitterness and antibacterial activities of constituents from *Evodia rutaecarpa*

**DOI:** 10.1186/s12906-017-1701-8

**Published:** 2017-03-29

**Authors:** Xiaoguang Liang, Bo Li, Fei Wu, Tingzhao Li, Youjie Wang, Qiang Ma, Shuang Liang

**Affiliations:** 1 Engineering Research Center of Modern Preparation Technology of TCM, Ministry of Education, Shanghai University of Traditional Chinese Medicine, 1200 Cailun Road, Shanghai, 201203 People’s Republic of China; 2 Amway (China) R & D Center Co., Ltd., Shanghai, 201203 People’s Republic of China; 3 Amway (China) Botanical R & D Center Co., Ltd., Wuxi, 214115 People’s Republic of China

**Keywords:** *Evodia rutaecarpa*, Electronic Tongue, Bitterness, Antibacterial activity

## Abstract

**Background:**

Bitter herbs are important in Traditional Chinese Medicine and the Electronic Tongue (e-Tongue) is an instrument that can be trained to evaluate bitterness of bitter herbs and their constituents. The aim of this research was to evaluate bitterness of limonoids and alkaloids from *Evodia rutaecarpa* to demonstrate that they are main bitter material basic of *E. rutaecarpa*.

**Methods:**

Nine compounds, including limonoids, indoloquinazoline alkaloids and quinolone alkaloids, were isolated, identified and analyzed by the e-Tongue. Additionally, the antibacterial activities of the nine compounds were evaluated against *E. coli* and *S. aureus*.

**Results:**

All the nine compounds had bitter taste and antibacterial activities to some extent. Among them, limonoids, which were the bitterest compounds, had greater antibacterial activities than alkaloids. And there is a positive correlation between bitter taste and antibacterial activities.

**Conclusions:**

It was confirmed in our study that limonoids, indoloquinazoline alkaloids and quinolone alkaloids are main bitter material basic of *E. rutaecarpa* based on two evaluation methods of e-Tongue and antibacterial experiment. In addition, the e-Tongue technique is a suitable new method to measure bitter degree in herbs.

**Electronic supplementary material:**

The online version of this article (doi:10.1186/s12906-017-1701-8) contains supplementary material, which is available to authorized users.

## Background

Bitter herbs are important in Traditional Chinese Medicine (TCM). According to the *Chinese Pharmacopoeia* 2010 edition, there are 177 species of bitter herbs, including many famous species, such as *Coptis chinensis, Evodia rutaecarpa* and *Rheum officinale* [1]. Based on the traditional theory and modern pharmacology, bitter herbs commonly possess widely pharmacological activities, including antibacterial, antitumor, regulating blood sugar level [2–5].

However, few studies have confirmed the correlation between the presence of bitter constituents in herbs and their pharmacological activities, mainly because there are no adequate methods to evaluate bitter constituents. Sensory panels are difficult to perform because of the possibility of health risks, and animal studies cannot be adequately controlled. The Electronic Tongue (e-Tongue) is an instrument that has been used to evaluate taste. In the past 20 years, the e-Tongue has been used to evaluate flavors in the food industry [6–8]. At the same time, pharmacy researchers began to use it in pharmaceutical research [9–12].

In a previous study, our laboratory evaluated the bitterness of berberine hydrochloride using the e-Tongue [13]. The study revealed that there were no differences between the e-Tongue data and the results obtained from a sensory taste panel, and e-Tongue is a suitable tool evaluating bitter constituents from TCM. Recently, we performed a study to evaluate *E. rutaecarpa* (Juss.) Benth., commonly referred to as “Wuzhuyu” in China. This herb, which has a spicy and bitter taste, has been used in the treatment of gastrointestinal disorders, abdominal pain, headache, and dysentery for thousands of years. Modern pharmacological studies have shown that the herb has antibacterial, anti-inflammatory, anti-infectious, and cytotoxic activities [14, 15]. Previous studies suggested that quinazolinedione alkaloids and limonoids are the most important effective components in *E. rutaecarpa* [16]. Among them*,* limonoids are representative natural bitter compounds.

In this paper, we describe the isolation, structural elucidation, bitterness evaluation using e-Tongue, and antibacterial activity testing of bitter constituents from *E. rutaecarpa*, and the correlation analysis between bitterness and antibacterial activities.

## Methods

### Plant materials and chemicals

*Evodia rutaecarpa* (Juss.) Benth. (No. 20130901) was purchased from Sichuan (China) and identified as *Evodia rutaecarpa* (Juss.) Benth. by Professor Zhi-li Zhao of the Shanghai University of Traditional Chinese Medicine (Shanghai, China). Materials for column chromatography included silica gel (10–40 μm; Huiyou Silical Gel Development Co. Ltd. Yantai, P. R. China), Sephadex LH-20 (40–70 μm; Amersham Pharmacia Biotech AB, Uppsala, Sweden), and reversed-phase C_18_ silica gel (50 μm; YMC, MA, U.S.A.). Acetonitrile (HPLC grade) was purchased from Merck KGaA (Darmstadt, Germany). Ultrahigh purified water used in this study was prepared in a Milli-Q water purification system (Millipore Corp., Billerica, MA, USA). All other reagents and chemicals, which were of analytical grade, were purchased from Sinopharm Chemical Reagent Company Ltd. (Shanghai, China).

### Bacterial strains and cultivation

*Escherichia coli* (CMCCB44113) and *Staphylococcus aureus* (CMCCB26003) were obtained from the Chinese National Center for Medical Culture Collections (CMCC, Beijing, China). Stock cultures in 25% glycerol were maintained at −80 °C. Bacterial strains were inoculated into lysogeny broth (LB) and grown for 6 h at 37 °C under constant shaking (165 rpm). Subsequently, the bacterial cultures were centrifuged at 3000 rpm for 5 min and washed three times with 10 mM phosphate-buffered solution (PBS, pH 7.4). The resulting pellets were re-suspended in PBS and inoculated into agar plates. Bacterial densities were determined using a scattered light turbidimeter; bacterial amount was derived from bacterial density.

### Equipments

An α-Astree liquid and taste analyzer (e-Tongue, Alpha Mos Inc) was coupled to seven taste sensors, an LS16 auto-sampler, and a reference electrode (Ag/AgCl, Alpha Mos Inc.). The system was equipped with a data acquisition and analysis software. The taste sensors included ZZ14601, AB11303, GA13207, BB14005, C13602, DA10905, and JE13101. NMR spectra were recorded in an Avance 500 NMR spectrometer with TMS as the standard. ESI-MS were measured in an Agilent LC/MSD Trap XCT mass spectrometer; HR-ESI-MS were measured in a Q-TOF micro mass spectrometer (Waters, USA). Optical rotations were acquired with a Perkin-Elmer 341 polarimeter, whereas IR spectra were recorded in a Bruker Vector 22 spectrometer with KBr pellets. Optical density was determined in an ELISA reader (CLX800-BioTek Instruments, USA).

### Extraction and Isolation of compounds **1**–**9**

Dried and powdered *E. rutaecarpa* fruits (6.0 kg) were extracted twice with methanol (48 L) under reflux for 2 h each time. Methanol was evaporated under vacuum, and the extract was suspended in water and successively partitioned with petroleum ether (3 × 10 L), CH_2_Cl_2_ (3 × 10 L), EtOAc (3 × 10 L), and n-butanol (3 × 10 L). The CH_2_Cl_2_ extract was subjected to CC on silica gel and eluted with gradient CH_2_Cl_2_-MeOH. The eluates were further subjected to on silica gel to give Compounds **1**, **2**, **3**, **4**, **5** and **8**. The EtOAc extract was also subjected to CC on silica gel and eluted successively with gradient CH_2_Cl_2_-MeOH mixtures of increasing polarity. The eluates were rechromatographed on ODS (CH_3_OH-H_2_O) followed by Sephadex LH-20 with CH_2_Cl_2_-MeOH (1:1) to give Compounds **6**, **7** and **9**.

### Chromatographic and mass spectrometric conditions

Chromatography was performed on an HPLC system (Thermo Finnigan, Thermo Corp., USA) equipped with a conditioned auto-sampler maintained at 10 °C and an Agilent ZORBAX Eclipse XDB-C_18_ column (4.6 × 150 mm, 5 μm, Agilent Corp., USA) maintained at 40 °C. A gradient elution was performed using acetonitrile (reagent A) and 0.1% acetic acid solution (reagent B) as the mobile phase: 10% reagent A from 0 to 0.5 min; 10 to 50% reagent A from 0.5 to 12 min, 50 to 95% reagent A from 12 to 17 min, and 95% reagent A for 1 min. Reagent A was reduced to 10% for column equilibration. The total cycle time was 23 min with a flow rate of 0.3 ml/min and an injection volume of 5.0 μl. A Finnigan LCQ DECA XP plus ion-trap Mass Spectrometer (Thermo Corp., USA) was connected to the HPLC system via an electrospray ionization (ESI) interface. The ESI source was operated in positive and negative ionization mode with a capillary voltage of 4.0 kV. The cone and desolvation gas flows were 10 L/h.

### Taste evaluation of compounds **1**–**9**

Compounds **1**–**9** were dissolved in 80 ml purified water (final concentration of the test solution, 9.6 mM). When the reference electrode and sensors were dipped into the test solution, a potentiometer difference between each individually coated sensor and the Ag/AgCl reference electrode was recorded by the e-Tongue. Each compound was analyzed for 120 s. The liquid sensors and the reference electrode were rinsed with purified water for 10 s in-between analyses. Using well-conditioned sensors, each compound was analyzed seven or eight times by a rotation procedure (i.e., the first round of compound measurements was completed before the next round of compound measurements was initiated) [13].

### Minimum inhibitory concentration (MIC) determination [17, 18]

The antibacterial activities of compounds **1**–**9** were assessed by the microdilution method [19]. Compounds **1**–**9** were dissolved in DMSO (10% of the final volume) and diluted with Mueller-Hinton broth to a concentration of 1 mg/ml. Further dilutions (1:3) were performed with the addition of culture broth, reaching concentrations that ranged from 0.037 to 1 mg/ml. An aliquot (200 μl) of each diluted compound was added to a 96-well plate. A growth control (containing Mueller-Hinton broth with DMSO, but without the compounds) and positive control drug (Kanamycin of 50 μg/ml) was added to the 96-well plate. The compounds and growth control were inoculated with 5 μl of the bacterial suspension and incubated at 36 °C for 24 h. Bacterial growth was assessed by optical density. MIC was defined as the lowest concentration of each compound that inhibited bacterial growth.

### Data analyses

Data were analyzed by principal component analysis (PCA) and partial least squares regression (PLS) [9, 11, 13]. PCA was used to estimate the largest and second largest relative contribution factors (i.e., PC1 and PC2) from the e-Tongue. The values were computed as a percentage by the Alpha MOS software. PC1 and PC2 explained the total variance of different samples and groups, respectively. Therefore, PCA assessed the bitterness of each isolated compound.

PLS was used to correlate the instrument data (sensor responses) with bitterness (bitterness intensity scores). On the PLS graph, the measurements corresponding to each compound (X-axis) were plotted against the instrumental data for each compound (Y-axis). A correlation was observed between the two data sets (solution and instrumental). The correlation coefficient (R^2^) of the PLS calibration curve should be equal or higher than 0.80. When the mixture sample was determined, the instrumental measurements could be obtained by the e-Tongue; bitterness (quantified as limonin) was calculated by PLS.

## Results and Discussion

### Phytochemistry of *E. rutaecarpa*

**Fig. 1:**
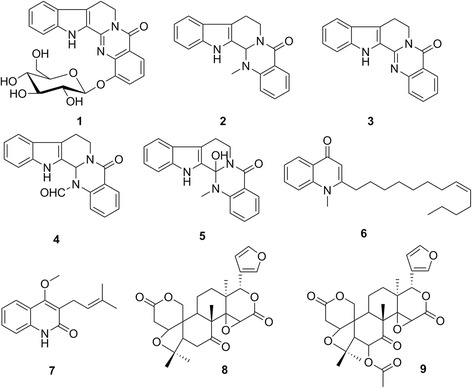
Structures of compounds **1**–**9**

The methanolic extract of *E. rutaecarpa* fruits was subjected to column chromatography over silica gel, RP-18, and Sephadex LH-20 in various solvent systems to afford five known indoloquinazoline alkaloids (**1**–**5**), two known quinazolinedione alkaloids (**6** and **7**), and two known limonoids (**8** and **9**). By comparing physical and spectroscopic data with reported data, the known compounds were identified as 1-O-*β*-D-glucopyranosylrutaecarpine (**1**) [20], evodiamine (**2**) [15], rutaecarpine (**3**) [21], 14-formyldihydro-rutaecarpine (**4**) [22], hydroxyevodiamine (**5**) [23], evocarpine (**6**) [24], 4-methoxy-3-(3-methylbut-2-enyl)-1*H*–quinolin-2-one (**7**) [25], limonin (**8**) [16], 6*β*-acetoxy-5-epilimonin (**9**, Fig. 1, Additional file 1) [26].

### Chromatographic and mass spectrometric results

**Fig. 2:**
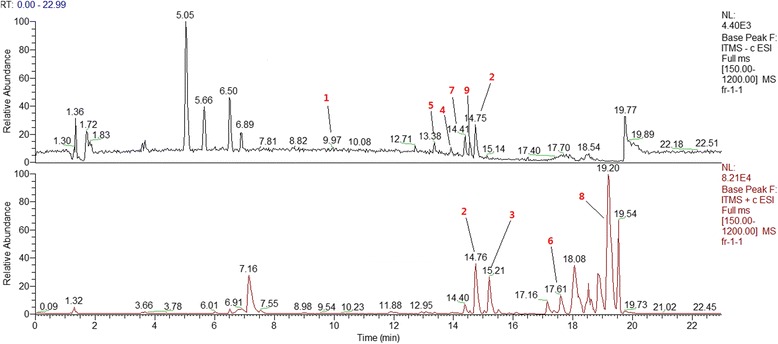
HPLC-MS chromatogram of *E. rutaecarpa* extract

The chromatographic and mass spectrometric results of *E. rutaecarpa* are shown in Fig. 2. The contents of compounds **1**–**9** are shown in Table 1. Among the compounds, compound **8** was the most predominant (2.0864%).

**Table 1:** Response values, bitterness relative to limonin, and contents of compounds **1**–**9**

Compounds	Response Values	Bitterness relative to limonin	Content (%)
1	3.691	0.377	0.0095
2	3.483	0.356	0.4852
3	4.499	0.459	0.3045
4	4.689	0.478	0.0360
5	4.154	0.424	0.1473
6	0.712	0.076	0.2355
7	2.081	0.214	0.2292
8	9.717	0.986	2.0864
9	8.901	0.904	0.1862

### Electronic tongue evaluation

The qualitative analysis result of PCA (Fig. 2) showed that the compounds in descending order of bitterness were **8** > **9** > **4** > **3** > **5** > **1** > **2** > **7** > **6**. The results revealed that limonoids are bitterer than indoloquinazoline alkaloids, which in turn are bitterer than quinolone alkaloids.

**Fig. 3:**
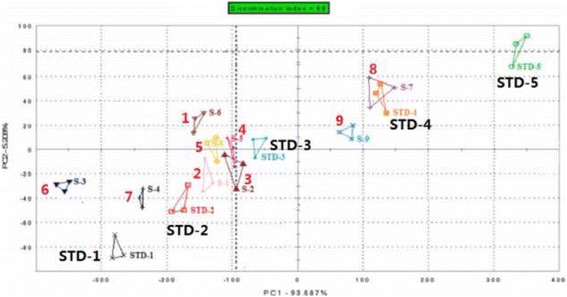
Principal component analysis (PCA) by e-Tongue

**Fig. 4:**
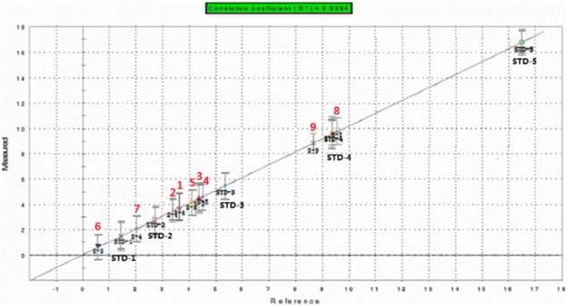
Partial least squares (PLS) analysis by e-Tongue

Limonin (**8**), a representative bitter natural compound, is a reference in PLS. Different concentrations of compound **8** (1.4, 2.8, 5.3, 9.4 and 16.5 mM) were evaluated by the e-Tongue (Fig. 3). There was a significant positive linear relationship for **8** at concentrations 1.4–16.5 mM (Y = 1.018 X-0.037, R^2^ = 0.999). The reference concentration was plotted on the X-axis; the response value of the e-Tongue was plotted on the Y-axis. The R.S.D. values for all samples were <5%. These results suggest that the sensor variation was insignificant. The response values of the other eight compounds generated linear equations. The relative bitterness of compounds **1**–**9** are shown in Fig. 4.

### Bacteriostatic activity

The result of antibacterial test (Table 2) suggested that all the nine compounds had antibacterial activities to some extent. Limonoids showed greater activities than alkaloids, and indoloquinazoline alkaloids had greater activities than quinolone alkaloids.

**Table 2:** Antibacterial activities of compounds **1**–**9** against *E. coli* and *S. aureus*

Compounds	*E. coli*				*S. aureus*			
	1 mg/ml	0.33 mg/ml	0.11 mg/ml	0.037 mg/ml	1 mg/ml	0.33 mg/ml	0.11 mg/ml	0.037 mg/ml
1	**−**	**+**	**+**	**+**	**−**	**+**	**+**	**+**
2	**−**	**−**	**+**	**+**	**−**	**+**	**+**	**+**
3	**−**	**−**	**+**	**+**	**−**	**+**	**+**	**+**
4	**−**	**+**	**+**	**+**	**+**	**+**	**+**	**+**
5	**−**	**+**	**+**	**+**	**+**	**+**	**+**	**+**
6	**−**	**+**	**+**	**+**	**+**	**+**	**+**	**+**
7	**−**	**+**	**+**	**+**	**+**	**+**	**+**	**+**
8	**−**	**−**	**−**	**+**	**−**	**−**	**−**	**+**
9	**−**	**−**	**−**	**+**	**−**	**−**	**+**	**+**

1. “+” represents bacteria can be observed in the experimental concentration; “**−**” represents bacteria can not be observed in the experimental concentration

2. Four compound concentration levels (1 mg/ml、0.33 mg/ml、0.11 mg/ml、0.037 mg/ml) represent four antibacterial activity intensities (0、1、2、3), respectively

Based on the contents of all constituents and bacteriostatic activity intensity, limonin (**8**) was the bitterest contributive compound of all the ones from *Evodia rutaecarpa*. Therefore, potential interactions amongst limonoids, indoloquinazoline alkaloids and quinolone alkaloids in bitterness contributions and antibacterial activity will be ahead the further direction of effort, such as synergistic, antagonistic, additive effect, etc.

In general, the modern electronic tongue technology with tremendous development potential play a more and more important role in the research of bitter herbs as well as wide applications in food industry and pharmaceutical analytics [6–12]. Yaroshenko [27] evaluated bitterness of eight various herbal medicine samples via three different approaches involving high-performance liquid chromatography coupled to UV detector, capillary electrophoresis coupled to UV detector and a potentiometric multisensor system – electronic tongue employing PLS regression and it was demonstrated that all three methods were able to be applied for quantitative assessment of bitterness in TCM with reasonable precision. Lin [28] constructed Robust Partial Least Squares (RPLS) model to evaluate the bitterness of a total of 35 TCM detoctions using an e-Tongue and that showed RPLS are capable of quantitatively characterizing bitterness. However, there is little study about application of electronic tongue to evaluate bitter constituents from TCM combined with pharmacological activity. Therefore, it is worth trying to explore and summarize for technological promotion of e-Tongue and pharmaceutical research from long-term perspective.

## Conclusions

Our findings suggest that limonoids are bitterer than indoloquinazoline alkaloids, and indoloquinazoline alkaloids are bitterer than quinolone alkaloids. Considering their contents in *E. rutaecarpa* given by the results of LC-MS, limonoids are the main contributors to the bitter taste of the herb. In addition, alkaloids, especially indoloquinazoline alkaloids, are other important bitter constituents of *E. rutaecarpa*.

Similarly, the result of antibacterial test suggested limonoids had greater antibacterial properties than alkaloids, and indoloquinazoline alkaloids had greater antibacterial properties than quinolone alkaloids. Therefore, there is a positive correlation between bitter taste and antibacterial activities. Further studies are needed to confirm these findings.

## Additional file

Additional file 1: The ^13^C and ^1^H NMR spectroscopic data of compounds 1–9. (DOC 58 kb)

## Abbreviations

COSY: Correlation spectroscopy
ELISA: Enzyme-linked immunosorbent assay
ESI: Electrospray ionization
ESI-MS: Electrospray ionization-mass spectrum
e-Tongue: Electronic Tongue
HMBC: Heteronuclear multiple-bond correlation
HPLC: High performance liquid chromatography
HR-ESI-MS: High resolution ESI-MS
HSQC: Heteronuclear single quantum coherence
IR: Infrared spectrometer
LB: Lysogeny broth
LC/MSD: Liquid chromatography/mass spectrometer detector
LC-MS: Liquid chromatography-mass spectrum
MIC: Minimum inhibitory concentration
NMR: Nuclear magnetic resonance
NOESY: Nuclear overhauser enhancement spectroscopy
PBS: Phosphate-buffered solution
PCA: Principal component analysis
PLS: Partial least squares
Q-TOF: Quadrupole-time-of-flight
R.S.D.: Relative standard deviation
TCM: Traditional Chinese Medicine
TMS: Tetramethylsilane
