# Supplementary material for: Bitterness and antibacterial activities of constituents from Evodia rutaecarpa
Source: BMC Complement Altern Med. 2017 Mar 29;17:180. doi: 10.1186/s12906-017-1701-8 (PMC5372309; doi:10.1186/s12906-017-1701-8)
Supplement: Additional file 1: — The 13C and 1H NMR spectroscopic data of compounds 1–9. (DOC 58 kb) [file 12906_2017_1701_MOESM1_ESM.doc]

**Bitterness and** **antibacterial activities of** **constituents from** ***Evodia rutaecarpa***

Xiaoguang Liang ^a^,†, Bo Li ^b^,†, Fei Wu ^a^, Tingzhao Li ^c^, Youjie Wang ^a^, Qiang Ma ^a^, Shuang Liang a,[[1]](#footnote-2)*

^a^ Engineering Research Center of Modern Preparation Technology of TCM, Ministry of Education, Shanghai University of Traditional Chinese Medicine, Shanghai 201203, PR China

^b^ Amway (China) R & D Center Co., Ltd., Shanghai 201203, PR China

^c^ Amway (China) Botanical R & D Center Co., Ltd., Wuxi 214115, PR China

The ^13^C and ^1^H NMR spectroscopic data of compounds **1**-**9**

Compound **1** (1-O-*β*-D-glucopyranosylrutaecarpine)

C_24_H_23_N_3_O_7_, ^13^C NMR (100 MHz, DMSO-*d_6_*) : *δ*_C_ 152.2 (C-1), 119.6 (C-2), 126.1 (C-3), 120.1 (C-4), 121.7 (C-4a), 160.3 (C-5), 40.8 (C-7), 18.8 (C-8), 117.6 (C-8a), 125.0 (C-8b), 120.0 (C-9), 119.8 (C-10), 124.7 (C-11), 112.4 (C-12), 138.4 (C-12a), 127.3 (C-13a), 144.0 (C-13a), 102.0 (C-1'), 73.4 (C-2'), 76.0 (C-3'), 69.8 (C-4'), 77.3 (C-5'), 60.8 (C-6')；

^1^H NMR (100 MHz, DMSO-*d_6_*) : *δ*_H_ 7.80 (1H, dd, *J* = 8.0, 1.2 Hz, H-2), 7.38 (1H, t, *J* = 8.0 Hz, H-3), 7.59 (1H, dd, *J* = 8.0, 1.2 Hz, H-4), 4.45 (2H, m, H-7), 3.19 (2H, t, *J* = 5.8, H-8), 7.67 (1H, d, *J* = 8.0 Hz, H-9), 7.11 (1H, t, *J* = 7.6 Hz, H-10), 7.30 (1H, t, *J* = 7.2 Hz, H-11), 7.56 (1H, d, *J* = 8.4 Hz, H-12), 5.00 (1H, d, *J* = 7.6 Hz, H-1'), 3.53 (1H, m, H-2'), 3.35 (1H, m, H-3'), 3.24 (1H, m, H-4'), 3.41 (1H, m, H-5'), 3.53 (1H, m, H-6') , 3.76 (1H, m, H-6'), 11.55 (1H, s, N-H), 5.32 (1H, d, *J* = 5.2 Hz, 2'-OH), 5.22 (1H, d, *J* = 5.2 Hz, 3'-OH), 5.10 (1H, d, *J* = 5.2 Hz, 4'-OH) , 4.74 (1H, t, *J* = 5.6 Hz, 6'-OH).

Compound **2** (evodiamine)

C_19_H_17_N_3_O, ^13^C NMR (100 MHz, CDCl_3_) : *δ*_C_ 128.3 (C-2), 68.9 (C-3), 39.6 (C-5), 20.1 (C-6), 113.6 (C-7), 126.3 (C-8), 129.0 (C-9), 123.9 (C-10), 123.0 (C-11), 111.3 (C-12), 136.7 (C-13), 150.6 (C-15), 122.2 (C-16), 133.1 (C-17), 120.0 (C-18), 118.9 (C-19), 123.6 (C-20), 164.8 (C-21);

^1^H NMR (400 MHz, CDCl_3_) : *δ*_H_ 2.52 (N-CH3, s), 2.97 (2H, m), 3.27 (1H, m), 4.87 (1H, m), 7.20 (4H, m), 7.48 (2H, m), 7.58 (2H, d, *J* = 7.6 Hz), 8.11 (1H, dd, *J* = 8.0, 1.6 Hz, H-19), 8.39 (1H, s, NH).

Compound **3** (rutaecarpine)

C_18_H_13_N_3_O, ^13^C NMR (100 MHz, CDCl_3_) : *δ*_C_ 127.2 (C-2), 145.1 (C-3), 41.1 (C-5), 19.6 (C-6), 118.5 (C-7), 125.6 (C-8), 120.6 (C-9), 120.1 (C-10), 125.6 (C-11), 112.1 (C-12), 138.3 (C-13), 147.3 (C-15), 126.4 (C-16), 134.4 (C-17), 126.2 (C-18), 127.0 (C-19), 121.1 (C-20), 161.5 (C-21);

^1^H NMR (400 MHz, CDCl_3_) : *δ*_H_ 9.76 (1H, s, NH), 4.58 (2H, t, *J* = 6.8 Hz, H-5), 3.22 (2H, t, *J* = 6.8 Hz, H-6), 7.63 (1H, brd, *J* = 8.0 Hz, H-9), 7.16 (1H, brdd, *J* = 8.0, 7.0Hz, H-10), 7.31 (1H, brdd, *J* = 8.0, 7.0Hz, H-11), 7.41 (1H, brd, *J* = 8.0 Hz, H-12), 7.61 (1H, brd, *J* = 8.4 Hz, H-16), 7.68 (1H, ddd, *J* = 8.4, 6.8, 1.2 Hz, H-17), 7.26 (1H, ddd, *J* = 8.4, 6.8, 1.2 Hz, H-18), 8.31 (1H, dd, *J* = 8.0, 0.8 Hz, H-19).

Compound **4** (14-formyldihydrorutaecarpine)

C_19_H_15_N_3_O_2_, ^13^C NMR (100 MHz, DMSO) : *δ*C 118.0 (C-1), 135.7 (C-2), 131.0 (C-3), 134.0 (C-4), 127.9 (C-4a), 163.2 (C-5), 43.6 (C-7), 19.1 (C-8), 111.7 (C-8a), 125.8 (C-8b), 118.1 (C-9), 119.0 (C-10), 121.8 (C-11), 111.6 (C-12), 136.9 (C-12a),132.9 (C-13a), 61.8 (C-13b), 137.0 (C-14a), 162.2 (N-CHO);

^1^H NMR (400 MHz, DMSO) : *δ*H11.14 (1H, s, N-H), 9.11 (1H, s, -CHO), 7.26 (1H, d, *J* = 7.5 Hz, H-1), 7.63 (1H, t , *J* = 8.0 Hz, H-2),6.96 (1H, t, *J* = 7.5 Hz, H-3), 7.89 (1H, d, *J* = 7.5 Hz, H-4), 4.70 (1H, dd, *J* = 5.0, 12.5 Hz, Ha-7), 3.62 (1H, td, *J* = 5.0, 12.5 Hz, Hb-7), 3.01 (1H, dddd, *J* = 1.5, 5.0, 12.5, 16.0 Hz, Ha-8), 2.65 (1H, dd, *J* = 5.0, 14 Hz, Hb-8), 7.58 (1H, d, *J* = 7.5 Hz, H-9), 7.06 (1H, d, *J* = 7.5 Hz, H-10), 7.27 (1H, t, *J* = 8.0 Hz, H-11), 7.38 (1H, d, *J* = 8.0 Hz, H-12).

Compound **5** (hydroxyevodiamine)

C_19_H_17_N_3_O_2_, ^13^C NMR (100 MHz, DMSO) : *δ***_C_** 120.9 (C-1), 138.7 (C-2), 134.0 (C-3), 137.0 (C-4), 130.9 (C-4a), 166.1 (C-5), 46.6 (C-7), 22.1 (C-8), 113.7 (C-8a), 127.9 (C-8b), 121.4 (C-9), 122.0 (C-10), 125.8 (C-11), 114.6 (C-12), 139.9 (C-12a), 136.0 (C-13a), 64.9 (C-13b), 137.0 (C-14a), 43.2 (N-CH_3_);

^1^H NMR (400 MHz, DMSO) : *δ***_H_** 11.12 (1H, s, N-H), 7.38 (1H, d, *J* = 7.5 Hz, H-1), 7.55 (1H, t , *J* = 8.0 Hz, H-2), 6.96 (1H, t, *J* = 7.5 Hz, H-3), 7.88 (1H, d, *J* = 7.5 Hz, H-4), 7.58 (1H, d, *J* = 7.5 Hz, H-9), 2.00 (N-CH_3_), 7.04 (1H, d, *J* = 7.5 Hz, H-10), 7.25 (1H, t, *J* = 8.0 Hz, H-11), 7.26 (1H, d, *J* = 8.0 Hz, H-12).

Compound **6** (evocarpine)

C_23_H_33_NO, ^13^C NMR (100 MHz, CDCl_3_) : *δ*_C_ 154.5 (C-2), 110.6 (C-3), 177.5 (C-4), 126.1 (C-4a), 126.1 (C-5), 122.9 (C-6), 131.7 (C-7), 115.2 (C-8), 141.8 (C-8a), 33.9 (N-CH_3_)34.4 (C-1'), 29.4 (C-2'), 29.1 (C-3'), 28.9 (C-4'), 28.6 (C-5'), 28.1 (C-6'), 26.9 (C-7'), 129.7 (C-8'), 129.3 (C-9'), 26.6 (C-10'), 31.7 (C-11'), 22.1 (C-12'), 13.8 (C-13');

^1^H NMR (400 MHz, CDCl_3_) : *δ*_H_ 6.03 (1H, s, H-3), 8.28 (1H, dd, *J* = 1.5, 8.0 Hz, H-5), 7.21 (1H, t, *J* = 8.0 Hz, H-6), 7.51 (1H, m, H-7), 7.33 (1H, d, *J* = 8.2 Hz, H-8), 3.56 (3H, s, N-CH_3_), 2.52 (2H, t, *J* = 8.0 Hz, H-1'), 1.49 (2H, m, H-2'), 1.31-1.21 (12H, m), 0.86 (3H, H-15).

Compound **7** (4-Methoxy-3-(3- methylbut-2-enyl)-1*H*-quinolin-2-one)

C_15_H_17_NO_2_, ^13^C NMR (100 MHz, CD_3_OD) : *δ*_C_ 166.5 (C-2), 122.7 (C-3), 163.9 (C-4), 118.4 (C-4a), 124.0 (C-5), 123.5 (C-6), 131.4 (C-7), 116.7 (C-8), 138.9 (C-8a), 24.3 (C-1'), 123.5 (C-2'), 133.4 (C-3'), 18.1, 25.9 (CH=C(CH_3_)_2_), 62.5 (OCH_3_);

^1^H NMR (600 MHz, CD_3_OD) : *δ*_H_ 7.77 (1H, dd, *J* = 7.8, 1.0 Hz, H-5), 7.49 (1H, ddd, *J* = 7.8, 6.6, 1.0 Hz, H-7), 7.34 (1H, dd, *J* = 7.8, 1.0 Hz, H-8), 7.25 (1H, ddd, *J* = 7.8, 6.6, 1.0 Hz, H-6), 5.23 (1H, t, *J* = 6.6 Hz, CH=CMe_2_), 3.92 (3H, s, OMe), 3.35 (2H, d, *J* = 6.6 Hz, CH2–CH=CMe_2_), 1.80 (3H, s, CH_3_), 1.68 (3H, s, CH_3_).

Compound **8** (limonin)

C_26_H_30_O_8_, ^13^C NMR (100 MHz, CDCl_3_) : *δ*_C_ 79.1 (C-1), 35.6 (C-2), 169.1 (C-3), 80.3 (C-4), 60.5 (C-5), 36.7 (C-6), 206.1 (C-7), 51.3 (C-8), 48.1 (C-9), 46.0 (C-10), 18.9 (C-11), 30.8 (C-12), 38.0 (C-13), 65.7 (C-14), 53.8 (C-15), 166.6 (C-16), 77.8 (C-17), 20.7 (C-18), 65.3 (C-19), 120.1 (C-20), 143.1 (C-21), 109.7 (C-22), 141.2 (C-23), 17.7 (C-24), 30.1 (C-25a), 21.4 (C-25b);

^1^H NMR (600 MHz, CDCl_3_) : *δ*_H_ 4.04 (1H, s, H-1), 2.68 (1H, dd, *J* = 17.0, 1.9 Hz, H-2α), 2.98 (1H, dd , *J* = 17.0, 4.0 Hz, H-2β), 2.23 (1H, dd , *J* = 15.0, 3.0 Hz, H-5), 2.47 (1H, dd, *J* = 14.0, 3.0 Hz, H-6α), 2.86 (1H, dd, *J* = 15.0, 14.0 Hz, H-6β), 2.55 (1H, dd, *J* = 12.0, 3.0 Hz, H-9), 4.04 (1H, s, H-15), 5.48 (1H, s, H-17), 1.17 (3H, s, H-18), 4.46 (1H, d, *J* = 13.0 Hz, H-19α), 4.77 (1H, d, *J* = 14.0 Hz, H-19β) , 7.41 (1H, s, H-21), 6.38 (1H, d, *J* = 1.2 Hz, H-22), 7.40 (1H, t, *J* = 2.0 Hz, H-23), 1.30 (3H, s, H-24), 1.18 (3H, s, H-25a), 1.07 (3H, s, H-25b).

Compound **9** (6*β*-acetoxy-5-epilimonin)

C_28_H_32_O_10_, ^13^C NMR (100 MHz, CDCl_3_) : δ_C_ 78.3 (C-1), 35.1 (C-2), 169.5 (C-3), 82.5 (C-4), 56.5 (C-5), 73.8 (C-6), 202.8 (C-7), 48.1 (C-8), 39.8 (C-9), 48.6 (C-10), 17.1 (C-11), 26.8 (C-12), 38.9 (C-13), 68.3 (C-14), 55.9 (C-15), 165.9 (C-16), 77.9 (C-17), 20.8 (C-18), 70.8 (C-19), 120.0 (C-20), 141.3 (C-21), 109.4 (C-22), 143.6 (C-23), 22.4 (C-28), 29.9 (C-29), 17.1 (C-30), 20.5 (CH_3_COO), 169.8 (CH_3_COO).

1. ^*^ Corresponding author at: Engineering Research Center of Modern Preparation Technology of TCM, Ministry of Education, Shanghai University of Traditional Chinese Medicine, 1200 Cailun Road, Shanghai 201203, PR China. Tel.: +86 21 51323094; Fax: +86 21 51323094.

   *E-mail address*: ls7312@163.com.

   † These authors have contributed equally to this work. [↑](#footnote-ref-2)
